# Genomic characteristics of quinolone resistance in colistin-resistant *Escherichia coli* isolates from community residents in Ecuador and Vietnam

**DOI:** 10.1093/jacamr/dlae151

**Published:** 2024-09-26

**Authors:** Hoa Thi Thanh Hoang, Mayumi Yamamoto, Yoshimasa Yamamoto

**Affiliations:** United Graduate School of Drug Discovery and Medical Information Sciences, Gifu University, Gifu, Japan; United Graduate School of Drug Discovery and Medical Information Sciences, Gifu University, Gifu, Japan; Health Administration Center, Gifu University, Gifu, Japan; United Graduate School of Drug Discovery and Medical Information Sciences, Gifu University, Gifu, Japan

Fluoroquinolones and quinolones are broad-spectrum antimicrobials widely used to treat various infectious diseases and are classified as critically important antimicrobials by the World Health Organization (https://apps.who.int/iris/handle/10665/325036). The abuse of quinolones in livestock can lead to the emergence and persistence of quinolone-resistant bacteria, which pose a significant public health threat.^1^ Additionally, widespread resistance to colistin, the highest-priority critical antimicrobial for human use, has been reported in developing countries.^2^ While emergence of resistance to quinolones and colistin is a major public health threat, the actual state of this phenomenon remains unclear, particularly in community settings.

Herein, we performed a genomic study on colistin-resistant *Escherichia coli* strains isolated from faecal samples of healthy residents in rural areas of Thai Binh province (Red River Delta), Vietnam (*n* = 37) and the cities of Santo Domingo (Pacific Coastal) and Puyo (Amazon), Ecuador (*n* = 47), during the period from 2017 to 2019,^3^ to elucidate the genetic background of quinolone resistance in these strains, focusing on mutations in the quinolone resistance-determining regions (QRDRs),^4^ and the presence of plasmid-mediated quinolone resistance (PMQR) genes.^5^ By analysing the complete genome of 84 colistin-resistant *E. coli* strains,^3^ we examined mutations in all quinolone resistance-associated QRDRs and PMQR genes, enabling a comprehensive analysis of the genetic structure of quinolone resistance in colistin-resistant *E. coli* in different geographical communities. Susceptibility to quinolones (ciprofloxacin [CIP] and nalidixic acid [NAL]) was assessed by the disc diffusion test, following the Clinical and Laboratory Standards Institute guidelines, as previously described.^6^

Mutations in QRDRs and the occurrence of PMQR genes were assessed using the DNA Data Bank of Japan Fast Annotation and Submission Tool v.1.6.0, ResFinder 4.1 and Prokka v1.14.5. The phylogenetic tree was based on single nucleotide polymorphism (SNP) data generated using PARsnp version 1.7.4, visualized on iTOLs. Maximum likelihood was constructed with 1000 bootstrap replicates using MEGA 11. The *χ*^2^ test (IBM SPSS Statistics v.20) was used for statistical analysis of differences in quinolone-resistant bacteria between the two countries.

Results of quinolone susceptibility and whole-genome analysis are summarized in Table 1; the original data used for the same are presented in Tables S1 and S2 (available as Supplementary data at *JAC-AMR* Online). All colistin-resistant *E. coli* strains from both countries harboured the colistin resistance gene *mcr-1*.^3^ The prevalence of quinolone resistance was 23.4% in Ecuadorian strains and 40.5% in Vietnamese strains; however, no statistically significant difference was observed between the two countries due to the limited number of strains examined.

**Table 1.** Quinolone resistance characteristics of community-isolated colistin-resistant *E. coli* strains

Country	No. of colistin-resistant strains tested (*n*)	No. of quinolone-resistant strains (% of colistin-resistant strains tested)	Resistance to		No. of strains with QRDR mutations associated with quinolone resistance (% of quinolone-resistant strains)				No. of strains with mutation(s) in QRDR (% of colistin-resistant strains tested)	No. of strains with PMQR (% of colistin-resistant strains tested)	
					*gyrA*	*gyrB*	*parC*	*parE*			
			CIP	NAL	S83L/D87N		A56T/S80I	I355T/S458A		*qnrB*	*qnrS*
Ecuador	47	11 (23.4)	3	11	6 (54.5)	0	6 (54.5)	1 (9.1)	21 (44.7)	19 (40.4)	4 (8.5)
Vietnam	37	15 (40.5)	8	15	10 (66.7)	0	13 (86.7)	1 (6.7)	31 (83.8)	0	23 (62.2)
*P* value^a^		0.092	0.184	–	0.53	–	0.068	0.819	<0.001	<0.001	<0.001

^a^ *P* value between Ecuador and Vietnam. –, no statistics.

CIP, ciprofloxacin; NAL, nalidixic acid.

Genomic analysis revealed that 83.8% of the 37 Vietnamese strains harboured QRDR mutations, compared to 44.7% of the 47 Ecuadorian strains, indicating a significantly lower mutation rate. Within the QRDR, mutations in *gyrA* (Ser83Leu and/or Asp87Asn) and *parC* (Ala56Thr and/or Ser80Ile), which are involved in quinolone resistance,^7,8^ were high in Vietnamese strains. A similar trend was observed in the 11 quinolone-resistant Ecuadorian strains. Mutations in *parE* (Ile355Thr and/or Ser458Ala), also associated with quinolone resistance,^7^ were less frequent, occurring in <9.1% of strains from Vietnam and Ecuador. Meanwhile, *gyrB* mutations, less commonly reported in relation to quinolone resistance, were rarely observed.

Examination of the correlation between QRDR mutations and quinolone resistance revealed that strains with *gyrA* and *parC* mutations, except for three strains, were resistant to both CIP and NAL, consistent with previous reports on the association of specific mutations with quinolone resistance.^9,10^ Notably, one Ecuadorian strain exhibited NAL resistance despite having neither the QRDR mutation nor the PMQR genes, suggesting an alternative resistance mechanism.

The mutation rates of QRDR genes were analysed regardless of quinolone resistance. Among the Vietnamese strains, *gyrA* had the highest mutation rate (56.8%), followed by *parC* (51.4%), *gyrB* (16.2%) and *parE* (10.8%) (Tables S1 and S2). In the Ecuadorian strains, the overall mutation rates were lower compared to that in the Vietnamese strains at 29.8% (*gyrA*), 25.5% (*parC*), 14.9% (*gyrB*) and 12.8% (*parE*). These results indicate that *gyrA* and *parC* are the most mutated genes and are closely linked to quinolone resistance in the community-isolated strains.

Utilizing whole genome sequencing and the aforementioned annotation methods, we identified PMQR genes and determined their locations. Among these isolates, nearly all detected PMQR genes were found to be located on plasmids, except for those in the LR-50 Ecuadorian strain (Table S2). The results showed that 62.2% of the Vietnamese strains exclusively harboured *qnrS*, compared to 8.5% of the Ecuadorian strains. Conversely, *qnrB* was the predominant PMQR gene in Ecuadorian strains (40.4%). Although the incompatibility types of PMQR gene-carrying plasmids were diverse, pO111 was identified as the primary carrier in Ecuadorian strains. The reasons for these differences in the prevalence of PMQR genes remain unclear. Nonetheless, the spread of bacteria within communities and the horizontal transfer of PMQR gene-carrying plasmids have been hypothesized to contribute to the distinctive PMQR gene profiles observed in community strains. Additionally, the observed variation in endemic PMQR genes suggests mechanisms distinct from those underlying QRDR mutation rates. Interestingly, no correlation was observed between PMQR genes and quinolone resistance in these strains.

SNP analysis of quinolone-resistant strains (Figure S1) revealed internal dissemination within the community for some Vietnamese isolates, determined as MLST 206. However, the majority did not exhibit strong phylogenetic similarities, suggesting that quinolone- and colistin-resistant *E. coli* are emerging as individual events. Nevertheless, the concurrent propagation of resistance to both colistin and quinolones within community settings poses a considerable threat to public health, given the clinical importance of these antibiotics.

## Supplementary Material

dlae151_Supplementary_Data

## Supplementary data

Tables S1 and S2 and Figure S1 are available as Supplementary data at *JAC-AMR* Online.
