# Supplementary material for: Genomic characteristics of quinolone resistance in colistin-resistant Escherichia coli isolates from community residents in Ecuador and Vietnam
Source: JAC Antimicrob Resist. 2024 Sep 26;6(5):dlae151. doi: 10.1093/jacamr/dlae151 (PMC11424991; doi:10.1093/jacamr/dlae151)
Supplement: dlae151_Supplementary_Data [file dlae151_supplementary_data.zip › Fig_S1.docx]

**A)**

**
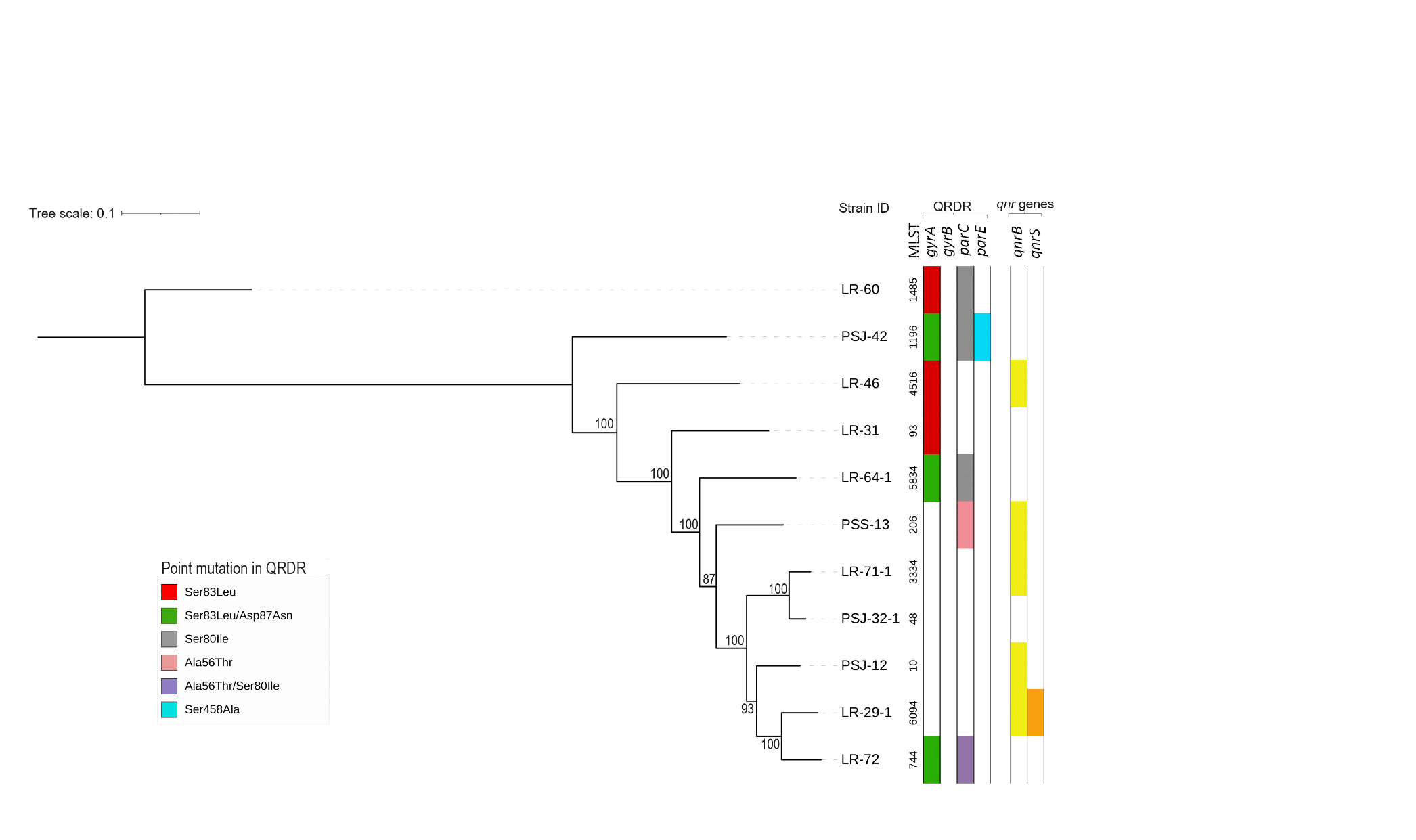
**

**B)**

**
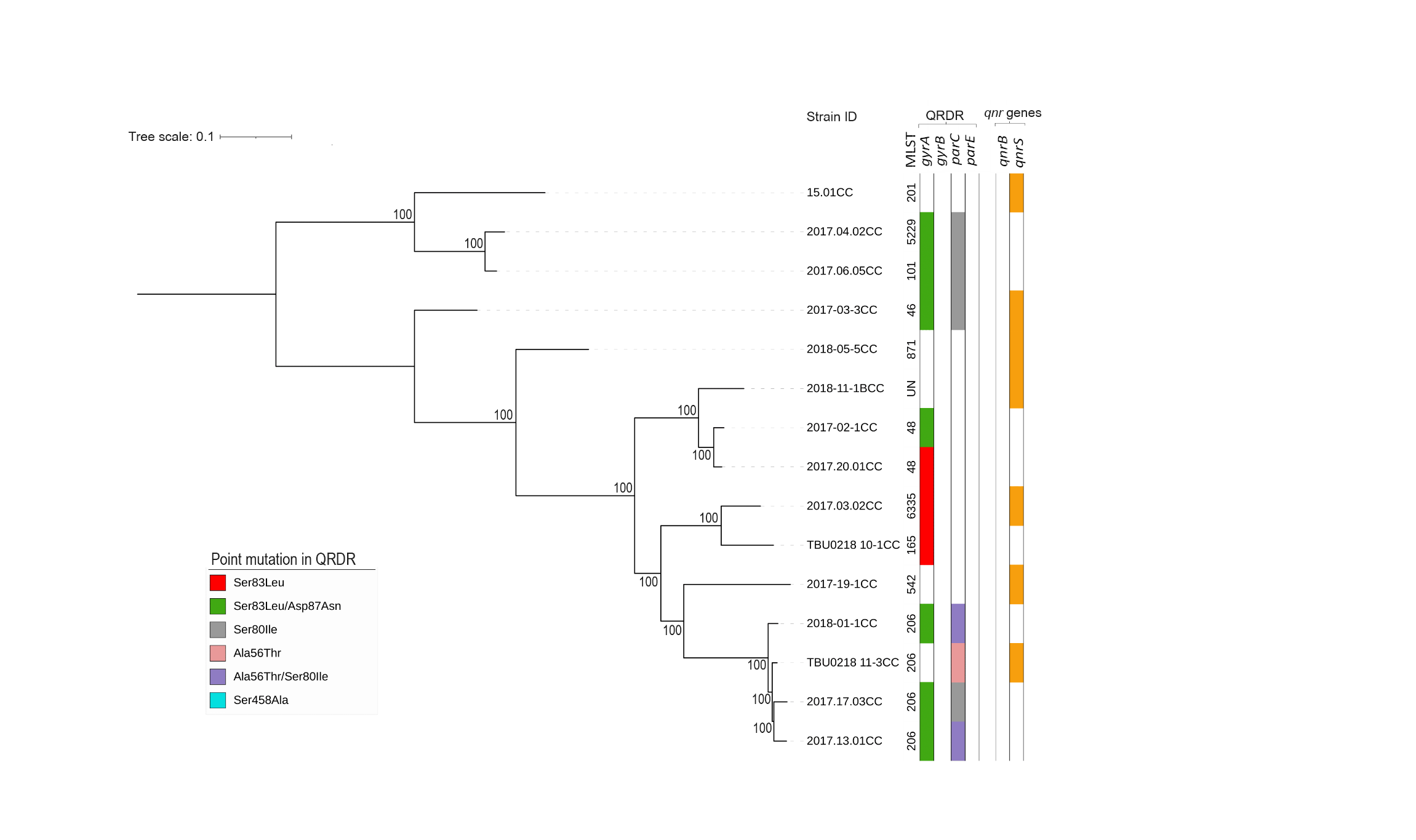
**

**Figure S1.** The SNP phylogenetic tree of quinolone-resistant *Escherichia coli* strains obtained from Ecuador (A) and Vietnam (B). Scale bar represents nucleotide substitutions per site.

SNP**,** single nucleotide polymorphism
